# Recombinant protein expression in *Escherichia coli*: advances and challenges

**DOI:** 10.3389/fmicb.2014.00172

**Published:** 2014-04-17

**Authors:** Germán L. Rosano, Eduardo A. Ceccarelli

**Affiliations:** ^1^Instituto de Biología Molecular y Celular de Rosario, Consejo Nacional de Investigaciones Científicas y TécnicasRosario, Argentina; ^2^Facultad de Ciencias Bioquímicas y Farmacéuticas, Universidad Nacional de RosarioRosario, Argentina

**Keywords:** recombinant protein expression, *Escherichia coli*, expression plasmid, inclusion bodies, affinity tags, *E. coli* expression strains

## Abstract

*Escherichia coli* is one of the organisms of choice for the production of recombinant proteins. Its use as a cell factory is well-established and it has become the most popular expression platform. For this reason, there are many molecular tools and protocols at hand for the high-level production of heterologous proteins, such as a vast catalog of expression plasmids, a great number of engineered strains and many cultivation strategies. We review the different approaches for the synthesis of recombinant proteins in *E. coli* and discuss recent progress in this ever-growing field.

## INTRODUCTION

There is no doubt that the production of recombinant proteins in microbial systems has revolutionized biochemistry. The days where kilograms of animal and plant tissues or large volumes of biological fluids were needed for the purification of small amounts of a given protein are almost gone. Every researcher that embarks on a new project that will need a purified protein immediately thinks of how to obtain it in a recombinant form. The ability to express and purify the desired recombinant protein in a large quantity allows for its biochemical characterization, its use in industrial processes and the development of commercial goods.

At the theoretical level, the steps needed for obtaining a recombinant protein are pretty straightforward. You take your gene of interest, clone it in whatever expression vector you have at your disposal, transform it into the host of choice, induce and then, the protein is ready for purification and characterization. In practice, however, dozens of things can go wrong. Poor growth of the host, inclusion body (IB) formation, protein inactivity, and even not obtaining any protein at all are some of the problems often found down the pipeline.

In the past, many reviews have covered this topic with great detail (Makrides, 1996; Baneyx, 1999; Stevens, 2000; Jana and Deb, 2005; Sorensen and Mortensen, 2005). Collectively, these papers gather more than 2000 citations. Yet, in the field of recombinant protein expression and purification, progress is continuously being made. For this reason, in this review, we comment on the most recent advances in the topic. But also, for those with modest experience in the production of heterologous proteins, we describe the many options and approaches that have been successful for expressing a great number of proteins over the last couple of decades, by answering the questions needed to be addressed at the beginning of the project. Finally, we provide a troubleshooting guide that will come in handy when dealing with difficult-to-express proteins.

## FIRST QUESTION: WHICH ORGANISM TO USE?

The choice of the host cell whose protein synthesis machinery will produce the precious protein will initiate the outline of the whole process. It defines the technology needed for the project, be it a variety of molecular tools, equipment, or reagents. Among microorganisms, host systems that are available include bacteria, yeast, filamentous fungi, and unicellular algae. All have strengths and weaknesses and their choice may be subject to the protein of interest (Demain and Vaishnav, 2009; Adrio and Demain, 2010). For example, if eukaryotic post-translational modifications (like protein glycosylation) are needed, a prokaryotic expression system may not be suitable (Sahdev et al., 2008). In this review, we will focus specifically on *Escherichia coli*. Other systems are described in excellent detail in accompanying articles of this series.

The advantages of using *E. coli* as the host organism are well known. (i) It has unparalleled fast growth kinetics. In glucose-salts media and given the optimal environmental conditions, its doubling time is about 20 min (Sezonov et al., 2007). This means that a culture inoculated with a 1/100 dilution of a saturated starter culture may reach stationary phase in a few hours. However, it should be noted that the expression of a recombinant protein may impart a metabolic burden on the microorganism, causing a considerable decrease in generation time (Bentley et al., 1990). (ii) High cell density cultures are easily achieved. The theoretical density limit of an *E. coli* liquid culture is estimated to be about 200 g dry cell weight/l or roughly 1 × 10^13^ viable bacteria/ml (Lee, 1996; Shiloach and Fass, 2005). However, exponential growth in complex media leads to densities nowhere near that number. In the simplest laboratory setup (i.e., batch cultivation of *E. coli* at 37°C, using LB media), <1 × 10^10^ cells/ml may be the upper limit (Sezonov et al., 2007), which is less than 0.1% of the theoretical limit. For this reason, high cell-density culture methods were designed to boost *E. coli* growth, even when producing a recombinant protein (Choi et al., 2006). Being a workhorse organism, these strategies arose thanks to the wealth of knowledge about its physiology. (iii) Rich complex media can be made from readily available and inexpensive components. (iv) Transformation with exogenous DNA is fast and easy. Plasmid transformation of *E. coli* can be performed in as little as 5 min (Pope and Kent, 1996).

## SECOND QUESTION: WHICH PLASMID SHOULD BE CHOSEN?

**FIGURE 1:**
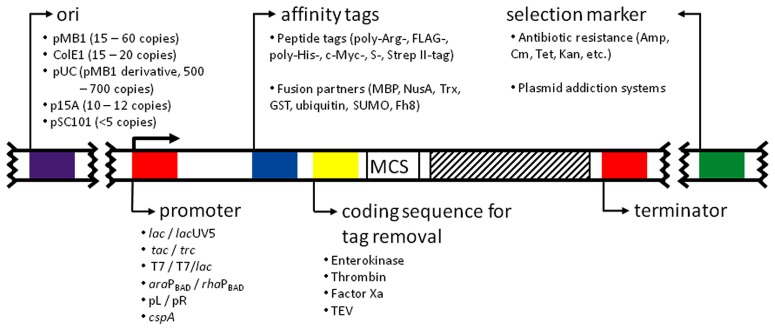
**Anatomy of an expression vector. The figure depicts the major features present in common expression vectors**. All of them are described in the text. The affinity tags and coding sequences for their removal were positioned arbitrarily at the N-terminus for simplicity. MCS, multiple cloning site. Striped patterned box: coding sequence for the desired protein.

The most common expression plasmids in use today are the result of multiple combinations of replicons, promoters, selection markers, multiple cloning sites, and fusion protein/fusion protein removal strategies (**Figure 1**). For this reason, the catalog of available expression vectors is huge and it is easy to get lost when choosing a suitable one. To make an informed decision, these features have to be carefully evaluated according to the individual needs.

### REPLICON

Genetic elements that undergo replication as autonomous units, such as plasmids, contain a replicon. It consists of one origin of replication together with its associated *cis*-acting control elements. An important parameter to have in mind when choosing a suitable vector is copy number. The control of copy number resides in the replicon (del Solar and Espinosa, 2000). It is logical to think that high plasmid dosage equals more recombinant protein yield as many expression units reside in the cell. However, a high plasmid number may impose a metabolic burden that decreases the bacterial growth rate and may produce plasmid instability, and so the number of healthy organisms for protein synthesis falls (Bentley et al., 1990; Birnbaum and Bailey, 1991). For this reason, the use of high copy number plasmids for protein expression by no means implies an increase in production yields.

Commonly used vectors, such as the pET series, possess the pMB1 origin (ColE1-derivative, 15–60 copies per cell; Bolivar et al., 1977) while a mutated version of the pMB1 origin is present in the pUC series (500–700 copies per cell; Minton, 1984). The wild-type ColE1 origin (15–20 copies per cell; Lin-Chao and Bremer, 1986; Lee et al., 2006) can be found in the pQE vectors (Qiagen). They all belong to the same incompatibility group meaning that they cannot be propagated together in the same cell as they compete with each other for the replication machinery (del Solar et al., 1998; Camps, 2010). For the dual expression of recombinant proteins using two plasmids, systems with the p15A ori are available (pACYC and pBAD series of plasmids, 10–12 copies per cell; Chang and Cohen, 1978; Guzman et al., 1995). Though rare, triple expression can be achieved by the use of the pSC101 plasmid. This plasmid is under a stringent control of replication, thus it is present in a low copy number (<5 copies per cell; Nordstrom, 2006). The use of plasmids bearing this replicon can be an advantage in cases where the presence of a high dose of a cloned gene or its product produces a deleterious effect to the cell (Stoker et al., 1982; Wang and Kushner, 1991). Alternatively, the use of the Duet vectors (Novagen) simplifies dual expression by allowing cloning of two genes in the same plasmid. The Duet plasmids possess two multiple cloning sites, each preceded by a T7 promoter, a *lac* operator and a ribosome binding site. By combining different compatible Duet vectors, up to eight recombinant proteins can be produced from four expression plasmids.

### PROMOTER

The staple in prokaryotic promoter research is undoubtedly the *lac* promoter, key component of the *lac* operon (Müller-Hill, 1996). The accumulated knowledge in the functioning of the system allowed for its extended use in expression vectors. Lactose causes induction of the system and this sugar can be used for protein production. However, induction is difficult in the presence of readily metabolizable carbon sources (such as glucose present in rich media). If lactose and glucose are present, expression from the *lac* promoter is not fully induced until all the glucose has been utilized. At this point (low glucose), cyclic adenosine monophosphate (cAMP) is produced, which is necessary for complete activation of the *lac* operon (Wanner et al., 1978; Postma and Lengeler, 1985). This positive control of expression is known as catabolite repression. In accordance, cAMP levels are low in cells growing in *lac* operon-repressing sugars, and this correlates with lower rates of expression of the *lac* operon (Epstein et al., 1975). Also, glucose abolishes lactose uptake because lactose permease is inactive in the presence of glucose (Winkler and Wilson, 1967). To achieve expression in the presence of glucose, a mutant that reduces (but does not eliminate) sensitivity to catabolite regulation was introduced, the *lac*UV5 promoter (Silverstone et al., 1970; Lanzer and Bujard, 1988). However, when present in multicopy plasmids, both promoters suffer from the disadvantage of sometimes having unacceptably high levels of expression in the absence of inducer (a.k.a. “leakiness”) due to titration of the low levels of the *lac* promoter repressor protein LacI from the single chromosomal copy of its gene (about 10 molecules per cell; Müller-Hill et al., 1968). Basal expression control can be achieved by the introduction of a mutated promoter of the *lacI* gene, called *lacI*^Q^, that leads to higher levels of expression (almost 10-fold) of LacI (Calos, 1978). The *lac* promoter and its derivative *lac*UV5 are rather weak and thus not very useful for recombinant protein production (Deuschle et al., 1986; Makoff and Oxer, 1991). Synthetic hybrids that combine the strength of other promoters and the advantages of the *lac* promoter are available. For example, the *tac* promoter consists of the -35 region of the *trp* (tryptophan) promoter and the -10 region of the *lac* promoter. This promoter is approximately 10 times stronger than *lac*UV5 (de Boer et al., 1983). Notable examples of commercial plasmids that use the *lac* or *tac* promoters to drive protein expression are the pUC series (*lac*UV5 promoter, Thermo Scientific) and the pMAL series of vectors (*tac* promoter, NEB).

The T7 promoter system present in the pET vectors (pMB1 ori, medium copy number, Novagen) is extremely popular for recombinant protein expression. This is not surprising as the target protein can represent 50% of the total cell protein in successful cases (Baneyx, 1999; Graumann and Premstaller, 2006). In this system, the gene of interest is cloned behind a promoter recognized by the phage T7 RNA polymerase (T7 RNAP). This highly active polymerase should be provided in another plasmid or, most commonly, it is placed in the bacterial genome in a prophage (λDE3) encoding for the T7 RNAP under the transcriptional control of a *lac*UV5 promoter (Studier and Moffatt, 1986). Thus, the system can be induced by lactose or its non-hydrolyzable analog isopropyl β-D-1-thiogalactopyranoside (IPTG). Basal expression can be controlled by *lac*I^Q^ but also by T7 lysozyme co-expression (Moffatt and Studier, 1987). T7 lysozyme binds to T7 RNAP and inhibits transcription initiation from the T7 promoter (Stano and Patel, 2004). In this way, if small amounts of T7 RNAP are produced because of leaky expression of its gene, T7 lysozyme will effectively control unintended expression of heterologous genes placed under the T7 promoter. T7 lysozyme is provided by a compatible plasmid (pLysS or pLysE). After induction, the amount of T7 RNAP produced surpasses the level of polymerase that T7 lysozyme can inhibit. The “free” T7 RNAP can thus engage in transcription of the recombinant gene. Yet another level of control lies in the insertion of a *lacO* operator downstream of the T7 promoter, making a hybrid T7/*lac* promoter (Dubendorff and Studier, 1991). All three mechanisms (tight repression of the *lac*-inducible T7 RNAP gene by *lac*I^Q^, T7 RNAP inhibition by T7 lysozyme and presence of a *lac*O operator after the T7 promoter) make the system ideal for avoiding basal expression.

The problem of leaky expression is a reflection of the negative control of the *lac* promoter. Promoters that rely on positive control should have lower background expression levels (Siegele and Hu, 1997). This is the case of the *araP*_BAD_ promoter present in the pBAD vectors (Guzman et al., 1995). The AraC protein has the dual role of repressor/activator. In the absence of arabinose inducer, AraC represses translation by binding to two sites in the bacterial DNA. The protein–DNA complex forms a loop, effectively preventing RNA polymerase from binding to the promoter. Upon addition of the inducer, AraC switches into “activation mode” and promotes transcription from the *ara* promoter (Schleif, 2000, 2010). In this way, arabinose is absolutely needed for induction.

Another widely used approach is to place a gene under the control of a regulated phage promoter. The strong leftward promoter (pL) of phage lambda directs expression of early lytic genes (Dodd et al., 2005). The promoter is tightly repressed by the λcI repressor protein, which sits on the operator sequences during lysogenic growth. When the host SOS response is triggered by DNA damage, the expression of the protein RecA is stimulated, which in turn catalyzes the self-cleavage of λcI, allowing transcription of pL-controlled genes (Johnson et al., 1981; Galkin et al., 2009). This mechanism is used in expression vectors containing the pL promoter. The SOS response (and recombinant protein expression) can be elicited by adding nalidixic acid, a DNA gyrase inhibitor (Lewin et al., 1989; Shatzman et al., 2001). Another way of activating the promoter is to control λcI production by placing its gene under the influence of another promoter. This two-stage control system has already been described for T7 promoter/T7 RNAP-based vectors. In the pLEX series of vectors (Life Technologies), the λcI repressor gene was integrated into the bacterial chromosome under the control of the *trp* promoter. In the absence of tryptophan, this promoter is always “on” and λcI is continuously produced. Upon addition of tryptophan, a tryptophan-TrpR repressor complex is formed that tightly binds to the *trp* operator, thereby blocking λcI repressor synthesis. Subsequently, the expression of the desired gene under the pL promoter ensues (Mieschendahl et al., 1986).

Transcription from all promoters discussed so far is initiated by chemical cues. Systems that respond to physical signals (e.g., temperature or pH) are also available (Goldstein and Doi, 1995). The pL promoter is one example. A mutant λcI repressor protein ( λcI^857^) is temperature-sensitive and is unstable at temperatures higher than 37°C. *E. coli* host strains containing the λcI^857^ protein (either integrated in the chromosome or into a vector) are first grown at 28–30°C to the desired density, and then protein expression is induced by a temperature shift to 40–42°C (Menart et al., 2003; Valdez-Cruz et al., 2010). The industrial advantage of this system lies in part in the fact that during fermentation, heat is usually produced and increasing the temperature in high density cultures is easy. On the other hand, genes under the control of the cold-inducible promoter *cspA* are induced by a downshift in temperature to 15°C (Vasina et al., 1998). This temperature is ideal for expressing difficult proteins as will be explained in another section. The pCold series of plasmids have a pUC118 backbone (a pUC18 derivative; Vieira and Messing, 1987) with the *cspA* promoter (Qing et al., 2004; Hayashi and Kojima, 2008). In the original paper, successful expression was achieved for more than 30 recombinant proteins from different sources, reaching levels as high as 20–40% of the total expressed proteins (Qing et al., 2004). However, it should be noted that in various cases the target proteins were obtained in an insoluble form.

### SELECTION MARKER

To deter the growth of plasmid-free cells, a resistance marker is added to the plasmid backbone. In the *E. coli* system, antibiotic resistance genes are habitually used for this purpose. Resistance to ampicillin is conferred by the *bla* gene whose product is a periplasmic enzyme that inactivates the β-lactam ring of β-lactam antibiotics. However, as the β-lactamase is continuously secreted, degradation of the antibiotic ensues and in a couple of hours, ampicillin is almost depleted (Korpimaki et al., 2003). Under this situation, cells not carrying the plasmid are allowed to increase in number during cultivation. Although not experimentally verified, selective agents in which resistance is based on degradation, like chloramphenicol (Shaw, 1983) and kanamycin (Umezawa, 1979), could also have this problem. For this reason, tetracycline has been shown to be highly stable during cultivation (Korpimaki et al., 2003), because resistance is based on active efflux of the antibiotic from resistant cells (Roberts, 1996).

The cost of antibiotics and the dissemination of antibiotic resistance are major concerns in projects dealing with large-scale cultures. Much effort has been put in the development of antibiotics-free plasmid systems. These systems are based on the concept of plasmid addiction, a phenomenon that occurs when plasmid-free cells are not able to grow or live (Zielenkiewicz and Ceglowski, 2001; Peubez et al., 2010). For example, an essential gene can be deleted from the bacterial genome and then placed on a plasmid. Thus, after cell division, plasmid-free bacteria die. Different subtypes of plasmid-addiction systems exist according to their principle of function: (i) toxin/antitoxin-based systems, (ii) metabolism-based systems, and (iii) operator repressor titration systems (Kroll et al., 2010). While this promising technology has been proved successful in large-scale fermentors (Voss and Steinbuchel, 2006; Peubez et al., 2010), expression systems based on plasmid addiction are still not widely distributed.

### AFFINITY TAGS

When devising a project where a purified soluble active recombinant protein is needed (as is often the case), it is invaluable to have means to (i) detect it along the expression and purification scheme, (ii) attain maximal solubility, and (iii) easily purify it from the *E. coli* cellular milieu. The expression of a stretch of amino acids (peptide tag) or a large polypeptide (fusion partner) *in tandem* with the desired protein to form a chimeric protein may allow these three goals to be straightforwardly reached (Nilsson et al., 1997).

Being small, peptide tags are less likely to interfere when fused to the protein. However, in some cases they may provoke negative effects on the tertiary structure or biological activity of the fused chimeric protein (Bucher et al., 2002; Klose et al., 2004; Chant et al., 2005; Khan et al., 2012). Vectors are available that allow positioning of the tag on either the N-terminal or the C-terminal end (the latter option being advantageous when a signal peptide is positioned at the N-terminal end for secretion of the recombinant protein, see below). If the three-dimensional structure of the desired protein is available, it is wise to check which end is buried inside the fold and place the tag in the solvent-accessible end. Common examples of small peptide tags are the poly-Arg-, FLAG-, poly-His-, c-Myc-, S-, and Strep II-tags (Terpe, 2003). Since commercial antibodies are available for all of them, the tagged recombinant protein can be detected by Western blot along expression trials, which is extremely helpful when the levels of the desired proteins are not high enough to be detected by SDS-PAGE. Also, tags allow for one-step affinity purification, as resins that tightly and specifically bind the tags are available. For example, His-tagged proteins can be recovered by immobilized metal ion affinity chromatography using Ni^2+^ or Co^2+^-loaded nitrilotriacetic acid-agarose resins (Porath and Olin, 1983; Bornhorst and Falke, 2000), while anti-FLAG affinity gels (Sigma-Aldrich) are used for capturing FLAG fusion proteins (Hopp et al., 1988).

On the other hand, adding a non-peptide fusion partner has the extra advantage of working as solubility enhancers (Hammarstrom et al., 2002). The most popular fusion tags are the maltose-binding protein (MBP; Kapust and Waugh, 1999), N-utilization substance protein A (NusA; Davis et al., 1999), thioredoxin (Trx; LaVallie et al., 1993), glutathione *S*-transferase (GST; Smith and Johnson, 1988), ubiquitin (Baker, 1996) and SUMO (Butt et al., 2005). The reasons why these fusion partners act as solubility enhancers remain unclear and several hypothesis have been proposed (reviewed in Raran-Kurussi and Waugh, 2012). In the case of MBP, it was shown that it possesses an intrinsic chaperone activity (Kapust and Waugh, 1999; Raran-Kurussi and Waugh, 2012). In comparison studies, GST showed the poorest solubility enhancement capabilities (Hammarstrom et al., 2006; Bird, 2011). NusA, MBP, and Trx display the best solubility enhancing properties but their large size may lead to the erroneous assessment of protein solubility (Costa et al., 2013). Indeed, when these tags are removed, the final solubility of the desired product is unpredictable (Esposito and Chatterjee, 2006). For these reasons, smaller tags with strong solubility enhancing effects are desirable. Recently, the 8-kDa calcium binding protein Fh8 from the parasite *Fasciola hepatica* was shown to be as good as or better than the large tags in terms of solubility enhancement. Moreover, the recombinant proteins maintained their solubility after tag removal (Costa et al., 2013). MBP and GST can be used to purify the fused protein by affinity chromatography, as MBP binds to amylose–agarose and GST to glutathione–agarose. MBP is present in the pMAL series of vectors from NEB and GST in the pGEX series (GE). A peptide tag must be added to the fusion partner-containing protein if an affinity chromatography step is needed in the purification scheme. MBP and GST bind to their substrates non-covalently. On the contrary, the HaloTag7 (Promega) is based on the covalent capture of the tag to the resin, making the system fast and highly specific (Ohana et al., 2009).

A different group of fusion tags are stimulus-responsive tags, which reversibly precipitate out of solution when subjected to the proper stimulus. The addition of β roll tags to a recombinant protein allows for its selective precipitation in the presence of calcium. The final products presented a high purity and the precipitation protocol only takes a couple of minutes (Shur et al., 2013). Another protein-based stimulus-responsive purification tags are elastin-like polypeptides (ELPs), which consist of tandem repeats of the sequence VPGXG, where X is Val, Ala, or Gly in a 5:2:3 ratio (Meyer and Chilkoti, 1999). These tags undergo an inverse phase transition at a given temperature of transition (*T*_t_). When the *T*_t_ is reached, the ELP–protein fusion selectively and reversibly precipitates, allowing for quick enrichment of the recombinant protein by centrifugation (Banki et al., 2005). Precipitation can also be triggered by adjusting the ionic strength of the solution (Ge et al., 2005). These techniques represent an alternative to conventional chromatography-based purification methods and can save production costs, especially in large-scale settings (Fong and Wood, 2010). The main characteristics of the tags mentioned in this section are outlined on **Table 1**.

**Table 1:** Main characteristics of protein fusion tags.

	Residues/Size (kDa)	Ligand/Matrix	Purification conditions	
***Peptide tags***	
Poly-Arg	Usually 5/0.80	Cation-exchange resin	NaCl linear gradient (0–400 mM)	
Poly-His	Usually 6/0.84	Ni^2+^-nitrilotriacetic acid-agarose	20–250 mM Imidazole/low pH	
FLAG	8/1.01	Anti-FLAG antibody immunodecorated agarose	2–5 mM EDTA	
Strep-tag II	8/1.06	Specially engineered streptavidin (Strep-Tactin)	2–25 mM desthiobiotin	
c-myc	11/1.20	Anti-myc antibody immunodecorated agarose	Low pH	
S-tag	15/1.75	S-protein (RNase A, residues 21–124) agarose	3 M guanidinium thiocyanate; 0.2 M potassium citrate buffer, pH 2 or 3 M MgCl_2_
**Fusion partners**^a^				**Solubility enhancement**^b^
Fh8	69/8.0	Ca^2+^-dependent binding to phenyl-Sepharose	10 mM EDTA	ND
Trx	109/11.7	4-amino phenylarsine oxide agarose (alternatively an affinity tag can be added)	5–1000 mM b-βmercaptoethanol	+++
SUMO	*ca*. 100/12.0	An affinity tag must be added (usually His-tag)		++++
BRT17 (β roll tag)	153/14.7		Precipitation in the presence of 25–75 mM Ca^2+^	ND
GST	211/26.0	Glutathione–agarose	10–20 mM reduced glutathione	+
HaloTag7	*ca.* 300/34.0	Chloroalkane ligand attached to agarose	A protease cleavage site is added between the tag and the protein for in-column cleavage	ND
MBP	396/*ca*. 42.5	Cross-linked amylose	10 mM maltose	+++
ELPs	550 (for 110 repeats)/*ca*. 47.0		Precipitation by temperature shifts and/or high concentrations of NaCl (>1.5 M)	ND
NusA	495/54.8	An affinity tag must be added (usually His-tag)		++

a Number of residues and size of fusion partners are approximate in some cases, as many variants exist. ^b^The grading in the solubility enhancement column is based on the results of Bird (Bird, 2011); ND, not determined in that study.

### TAG REMOVAL

If structural or biochemical studies on the recombinant protein are needed, then the fusion partner must be eliminated from the recombinant protein. Peptide tags should be removed too because they can interfere with protein activity and structure (Wu and Filutowicz, 1999; Perron-Savard et al., 2005), but they can be left in place even for crystallographic studies (Bucher et al., 2002; Carson et al., 2007). Tags can be eliminated by either enzymatic cleavage or chemical cleavage.

In the case of tag removal by enzyme digestion, expression vectors possess sequences that encode for protease cleavage sites downstream of the gene coding for the tag. Enterokinase, thrombin, factor Xa and the tobacco etch virus (TEV) protease have all been successfully used for the removal of peptide tags and fusion partners (Jenny et al., 2003; Blommel and Fox, 2007). Choosing among the different proteases is based on specificity, cost, number of amino acids left in the protein after cleavage and ease of removal after digestion (Waugh, 2011). Enterokinase and thrombin were popular in the past but the use of His-tagged TEV has become an everyday choice due to its high specificity (Parks et al., 1994), it is easy to produce in large quantities (Tropea et al., 2009) and leaves only a serine or glycine residue (or even the natural N-terminus) after digestion (Kapust et al., 2002).

As the name implies, in chemical cleavage the tag is removed by treatment of the fusion protein with a chemical reagent. The advantages of using chemicals for this purpose are that they are easy to eliminate from the reaction mixture and are cheap in comparison with proteolytic enzymes, which makes them an attractive choice in the large-scale production of recombinant proteins (Rais-Beghdadi et al., 1998). However, the reaction conditions are harsh, so their use is largely restricted to purified recombinant proteins obtained from IBs. They also often cause unwanted protein modifications (Hwang et al., 2014). The most common chemical cleavage reagent is cyanogen bromide (CNBr). CNBr cleaves the peptide bond C-terminal to methionine residues, so this amino acid should be present between the tag and the protein of interest (Rais-Beghdadi et al., 1998). Also, the target protein should not contain internal methionines. CNBr cleavage can be performed in common denaturing conditions (6 M guanidinium chloride) or 70% formic acid or trifluoroacetic acid (Andreev et al., 2010). Other chemical methods for protein cleavage can be found in Hwang et al. (2014).

## THIRD QUESTION: WHICH IS THE APPROPRIATE HOST?

A quick search in the literature for a suitable *E. coli* strain to use as a host will yield dozens of possible candidates. All of them have advantages and disadvantages. However, something to keep in mind is that many are specialty strains that are used in specific situations. For a first expression screen, only a couple of *E. coli* strains are necessary: BL21(DE3) and some derivatives of the K-12 lineage.

The history of the BL21 and BL21(DE3) strains was beautifully documented in Daegelen et al. (2009) and we recommend this article to the curious. BL21 was described by Studier in 1986 after various modifications of the B line (Studier and Moffatt, 1986), which in turn Daegelen et al. (2009) traced back to d’Herelle. A couple of genetic characteristics of BL21 are worthy of mention. Like other parental B strains, BL21 cells are deficient in the Lon protease, which degrades many foreign proteins (Gottesman, 1996). Another gene missing from the genome of the ancestors of BL21 is the one coding for the outer membrane protease OmpT, whose function is to degrade extracellular proteins. The liberated amino acids are then taken up by the cell. This is problematic in the expression of a recombinant protein as, after cell lysis, OmpT may digest it (Grodberg and Dunn, 1988). In addition, plasmid loss is prevented thanks to the *hsd*SB mutation already present in the parental strain (B834) that gave rise to BL21. As a result, DNA methylation and degradation is disrupted. When the gene of interest is placed under a T7 promoter, then T7 RNAP should be provided. In the popular BL21(DE3) strain, the λDE3 prophage was inserted in the chromosome of BL21 and contains the T7 RNAP gene under the *lac*UV5 promoter, as was explained earlier.

The BL21(DE3) and its derivatives are by far the most used strains for protein expression. Still, there are reports where the K-12 lineage is used for this purpose. The AD494 and Origami^TM^ (Novagen) strains are *trx*B (thioredoxin reductase) mutants, so disulfide bond formation in the cytoplasm is enhanced (the Origami strain also lacks the glutathione reductase gene; Derman et al., 1993). Another widely used strain from the K-12 repertoire is HMS174, a *recA* mutant (Campbell et al., 1978). This mutation has a positive effect on plasmid stability (Marisch et al., 2013). Plasmid multimer formation, an important cause of instability, relies on the recombination system of *E. coli* (Summers et al., 1993). All three strains have their λDE3-containing derivative (available at Novagen) so the T7 RNAP system can be used.

## FOURTH QUESTION: WHICH IS THE COMBINATION FOR SUCCESS?

At this point, it should be pretty clear that the number of options when designing an expression system is considerably high. Choosing the perfect combination is not possible *a priori*, so multiple conditions should be tested to obtain the desired protein. If the project demands expressing two protein constructs, cloned in six different expression vectors, each transformed in three different expression strains, then you are in for 36 expression trials. This number may be even higher when other variables are taken into account. This trial-and-error and time consuming pilot study can be made faster if micro-expression trials are performed before scale-up. Small-scale screens can be performed in 2-ml tubes or 96-well plates (Shih et al., 2002). High throughput protocols adapting automatic liquid handling robots have been described, making it possible for a single person to test more than 1000 culture conditions within a week.

## TROUBLESHOOTING RECOMBINANT PROTEIN PRODUCTION

This section of the review covers different strategies for optimizing recombinant protein production in *E. coli*. Even after careful selection of plasmid and host, it cannot be predicted if the protein will be obtained in high amounts and in a soluble active form. Various situations that impede reaching that goal can be encountered, which unfortunately happen very often. Many things to try in each case are discussed in the following paragraphs and, for convenience of the readers; a summary is included in **Table 2**.

**Table 2:** Strategies for overcoming common problems during recombinant protein expression in *E. coli*.

Problem	Possible explanation	Solutions
No or low expression	Protein may be toxic before induction	Control basal induction:

		• add glucose when using expression vectors containing *lac*-based promoters
		• use defined media with glucose as source of carbon
		• use pLysS/pLysE bearing strains in T7-based systems
		• use promoters with tighter regulation
		Lower plasmid copy number
	Protein may be toxic after induction	Control level of induction:
		• Tuneable promoters
		• Use strains that allow control of induction [Lemo21(DE3) strain] or *lacY*^-^ strains (Tuner^TM^)
		Lower plasmid copy number
		Use strains that are better for the expression of toxic proteins (C41 or C43)
		Direct protein to the periplasm
	Codon bias	Optimize codon frequency in cDNA to better reflect the codon usage of the host
		Use codon bias-adjusted strains
		Increase biomass:
		• Try new media formulations
		• Provide good aeration and avoid foaming
Inclusion body formation	Incorrect disulfide bond formation	Direct protein to the periplasm
		Use *E. coli* strains with oxidative cytoplasmic environment
	Incorrect folding	Co-express molecular chaperones
		Supplement media with chemical chaperones and cofactors
		Remove inducer and add fresh media
		Lower production rate:
		• Lower temperature. If possible, use strains with cold-adapted chaperones
		• Tune inducer concentration
	Low solubility of the protein	Fuse desired protein to a solubility enhancer (fusion partners)
	An essential post translational modification is needed	Change microorganism
Protein inactivity	Incomplete folding	Lower temperature
		Monitor disulfide bond formation and allow further folding* in vitro*
	Mutations in cDNA	Sequence plasmid before and after induction. If mutations are detected, the protein may be toxic.
		Use a *recA*^-^ strain to ensure plasmid stability
		Transform *E. coli* before each expression round

### NO OR LOW PRODUCTION

This situation may be regarded as the worst case scenario. When the protein of interest cannot be detected through a sensitive technique (e.g., Western blot) or it is detected but at very low levels (less than micrograms per liter of culture), the problem often lies in a harmful effect that the heterologous protein exerts on the cell (Miroux and Walker, 1996; Dumon-Seignovert et al., 2004).

#### Protein toxicity

The problem of protein toxicity may arise when the recombinant protein performs an unnecessary and detrimental function in the host cell. This function interferes with the normal proliferation and homeostasis of the microorganism and the visible result is slower growth rate, low final cell density, and death (Doherty et al., 1993; Dong et al., 1995).

As a first measure, cell growth should be monitored before induction. If the growth rate of the recombinant strain is slower compared to an empty-vector bearing strain then two causes may explain the phenotype: gene toxicity and basal expression of the toxic mRNA/protein. Gene toxicity will not be discussed here and the review of Saida et al. (2006) is recommended.

The control of basal synthesis was covered in some detail in Section “Promoter.” As stated, the expression of LacI from *lacI* or *lacI*^Q^ represses transcription of *lac*-based promoters. For high copy number plasmids (>100 copies per cell), *lacI*^Q^ should be cloned in the expression vector. The pQE vectors from Qiagen utilize two *lac* operator sequences to increase control of the T5 promoter, which is recognized by the *E. coli* RNA polymerase (see The QIAexpressionist^TM^ manual from Qiagen). A tighter control can be achieved by the addition of 0.2–1% w/v glucose in the medium as rich media prepared with tryptone or peptone may contain the inducer lactose (Studier, 2005). Another option could be to prepare defined media using glucose as a source of carbon. In T7-based promoters, leaky expression is avoided by co-expression of T7 lysozyme from the pLysS or pLysE plasmids (see above). Use of lower copy number plasmids containing tightly regulated promoters (like the *araP_BAD_* promoter) is suggested. An interesting case of copy number control is the one employed in pETcoco vectors (Novagen). These plasmids possess two origins of replication. The *ori*S origin and its control elements maintain pETcoco at one copy per cell (Wild et al., 2002). However, the TrfA replicator activates the medium-copy origin of replication (*ori*V) and amplification of copy number is achieved (up to 40 copies per cell). The *trf*A gene is on the same vector and is under control of the *araP_BAD_* promoter, so copy number can be controlled by arabinose (Wild et al., 2002).

After control of basal expression, the culture should grow well until the proper time of induction. At this moment, if the protein is toxic, cell growth will be arrested. In many cases, the level of toxicity of a protein becomes apparent when a certain threshold of host tolerance is reached and exceeded. In such situations, the level of expression should be manipulated at will. Tunable expression can be achieved using the Lemo21(DE3) strain. This strain is similar to the BL21(DE3)pLysS strain, however, T7 lysozyme production from the *lysY* gene is under the tunable promoter *rhaP_BAD_* (Wagner et al., 2008). At higher concentrations of the sugar L-rhamnose, more T7 lysozyme is produced, less active T7 RNAP is present in the cell and less recombinant protein is expressed. Trials using L-rhamnose concentrations from 0 to 2,000 μM should be undertaken to find the best conditions for expression. By contrast, dose-dependent expression when using IPTG as inducer is not possible since IPTG can enter the cell by active transport through the Lac permease or by permease-independent pathways (Fernandez-Castane et al., 2012). Since expression of Lac permease is heterogeneous and the number of active permeases in each cell is highly variable, protein expression does not respond predictably to IPTG concentration. The Tuner^TM^ (DE3) strain (Novagen) is a BL21 derivative that possesses a lac permease (*lacY*) mutation that allows uniform entry of IPTG into all LacY^-^ cells in the population, which produces a concentration-dependent, homogeneous level of induction (Khlebnikov and Keasling, 2002). In the same line of thought, an *E. coli* strain was constructed by exchanging the wild-type operator by the derivative *lacO^c^*, thus converting the *lac* operon into a constitutive one. This modification avoids the transient non-genetic LacY^-^ phenotype of a fraction of the cells, allowing uniform entry of the inducer lactose. A second modification (*gal*^+^) permits the full utilization of lactose as an energy source (Menzella et al., 2003).

A word of caution needs to be said in regard to “tunable promoters” that are inducible by sugars (lactose, arabinose, rhamnose). In the case of the *araP_BAD_* promoter, the yields of the target protein can be reproducibly increased over a greater than 100-fold range by supplementing the culture with different sub-maximal concentrations of arabinose (Guzman et al., 1995). This led to the erroneous belief that within each cell, the level of recombinant protein synthesis can be manipulated at will. However, it was shown that the range in protein expression arises from the heterogeneity in the amount of active sugar permeases in each cell, as was also explained for LacY (Siegele and Hu, 1997). So, even though the final protein yield can be controlled, the amount of protein per cell is widely variable, with cells producing massive amounts of protein and others not producing any protein at all. This can be a nuance, since in the case of toxic products; the subpopulation of cells with high-level synthesis may perish (Doherty et al., 1993; Dong et al., 1995).

Some *E. coli* mutants were specifically selected to withstand the expression of toxic proteins. The strains C41(DE3) and C43(DE3) were found by Miroux and Walker (1996) in a screen designed to isolate derivatives of BL21(DE3) with improved membrane protein overproduction characteristics. It was recently discovered that the previously uncharacterized mutations which prevent cell death during the expression of recombinant proteins in these strains lie on the *lac*UV5 promoter. In BL21(DE3) cells, the *lac*UV5 promoter drives the expression of the T7 RNAP, but in the Walker strains two mutations in the -10 region revert the *lac*UV5 promoter back into the weaker wild-type counterpart. This leads to a lesser (and perhaps more tolerable for the cell) level of synthesis (Wagner et al., 2008).

Another solution could be to remove the protein from the cell. Secretion to the periplasm or to the medium is sometimes the only way to produce a recombinant protein (Mergulhao et al., 2005; de Marco, 2009). The first option for expression in the periplasm is the post-translational Sec-dependent pathway (Georgiou and Segatori, 2005). Routing to the extracytoplasmatic space is achieved by fusing the recombinant protein to a proper leader peptide. The signal peptides of the following proteins are widely used for secretion: Lpp, LamB, LTB, MalE, OmpA, OmpC, OmpF, OmpT, PelB, PhoA, PhoE, or SpA (Choi and Lee, 2004). The co-translational translocation machinery based on the SRP (signal recognition particle) pathway can also be used. SRP recognizes its substrates by the presence of a hydrophobic signal sequence located in the N-terminal end. Following interaction with the membrane receptor FtsY, the complex of nascent chain and ribosome is transferred to the SecYEG translocase (Valent et al., 1998). The signal sequence of disulfide isomerase I (DsbA) has been used to target recombinant proteins to the periplasm via the SRP pathway. Notable examples of recombinant proteins secreted though this system include thioredoxin (Schierle et al., 2003) and the human growth hormone (Soares et al., 2003).

#### Codon bias

Codon bias arises when the frequency of occurrence of synonymous codons in the foreign coding DNA is significantly different from that of the host. At the moment of full synthesis of the recombinant protein, depletion of low-abundance tRNAs occurs. This deficiency may lead to amino acid misincorporation and/or truncation of the polypeptide, thus affecting the heterologous protein expression levels (which will be low at best) and/or its activity (Gustafsson et al., 2004). To check if codon bias could be an issue when expressing a recombinant protein, a large number of free online apps detect the presence of rare codons in a given gene when *E. coli* is used as a host (molbiol.ru/eng/scripts/01_11.html, genscript.com/cgibin/tools/rare_codon_analysis, nihserver.mbi.ucla.edu/RACC/, just to name a few). Rare codons were defined as codons used by *E. coli* at a frequency <1% (Kane, 1995). For example, the AGG codon (Arg) is used in *E. coli* at a frequency of <0.2%, but it is not rare in plant mRNAs where it can reach frequencies >1.5%.

Two strategies for solving codon usage bias have been used: codon optimization of the foreign coding sequence or increasing the availability of underrepresented tRNAs by host modification (Sorensen and Mortensen, 2005). The rationale behind codon usage optimization is to modify the rare codons in the target gene to mirror the codon usage of the host (Burgess-Brown et al., 2008; Welch et al., 2009; Menzella, 2011). The amino acid sequence of the encoded protein must not be altered in the process. This can be done by site-directed silent mutagenesis or resynthesis of the whole gene or parts of it. Codon optimization by silent mutagenesis is a cumbersome and expensive process, so is not very useful when many recombinant proteins are needed. On the other hand, gene synthesis by design is not a trivial issue since it requires choosing the best sequence from a vast number of possible combinations (Gustafsson et al., 2004). The simplest approach is to replace all instances of a given amino acid in the target gene by the most abundant codon of the host, a strategy called “one amino acid-one codon.” More advanced algorithms, which employ several other optimization parameters such as codon context and codon harmonization, have been described (Gao et al., 2004; Supek and Vlahovicek, 2004; Jayaraj et al., 2005; Angov et al., 2011). Some are freely available as web servers or standalone software. For a comprehensive list, please refer to Puigbo et al. (2007).

Correcting codon usage is a tricky situation. The “one amino acid-one codon” strategy disregards factors other than codon rarity that influence protein expression levels. For example, in bacterial genes enriched in rare codons at the N-terminus, protein expression is actually improved. The cause lies not in codon rarity *per se* but in the reduction of RNA secondary structure (Goodman et al., 2013). In addition, a recent report has shown that high levels of protein production are mainly (but not only) determined by the decoding speed of the open reading frame (i.e., the time it takes for a ribosome to translate an mRNA), especially if “fast” codons are located at the 5′-end of the mRNA (Chu et al., 2014). This causes a fast ribosome clearance at the initiation site, so that new recruited ribosomes encounter a free start codon and can engage in translation. Finally, some codon combinations can create Shine–Dalgarno-like structures that cause translational pausing by hybridization between the target mRNA and the 16S rRNA of the translating ribosome (Li et al., 2012). Translational pausing along the mRNA has a beneficial effect in protein folding, as it allows for the newly synthesized chain to adopt a well-folded intermediate conformation (Thanaraj and Argos, 1996; Oresic and Shalloway, 1998; Tsai et al., 2008; Yona et al., 2013). All of this new evidence in translational control mechanisms poses a challenge in the rational design of synthetic genes. Newer algorithms should account for 5′ RNA structure, presence of strategically located Shine–Dalgarno-like motifs, ribosome clearance rates at the initiation site and presence of slowly translated regions that are beneficial in co-translational folding.

On the other hand, when the cell is producing massive amounts of proteins (as in the case of recombinant expression of heterologous genes), charged tRNA availability for rare codons does become the major determinant of the levels of produced protein (Pedersen, 1984; Li et al., 2012). Low-abundance tRNA depletion causes ribosome stalling and its subsequent detachment from the RNA strand and thus, failure to generate a full-length product (Buchan and Stansfield, 2007). Several strains carrying plasmids containing extra copies of problematic tRNAs genes can be used to circumvent this issue. The BL21(DE3)CodonPlus strain (Stratagene) contains the pRIL plasmid (p15A replicon, which is compatible with the ColE1 and ColE1-like origins contained in most commonly used expression vectors), which provides extra genes for the tRNAs for AGG/AGA (Arg), AUA (Ile), and CUA (Leu). BL21(DE3)CodonPlus-RP (Stratagene) corrects for the use of AGG/AGA (Arg) and CCC (Pro). The Rosetta(DE3) strains (Novagen) are Tuner^TM^ derivatives containing the pRARE plasmid (p15A replicon), supplying tRNAs for all the above-mentioned codons plus GGA (Gly). It should be noted that the use of these strains often improves the levels of protein production but sometimes can cause a decrease in protein solubility. We have found that proteins with higher than 5% content of RIL codons (AGG/AGA, AUA, and CUA) are less soluble when expressed in the CodonPlus strain. In this host, the translational pauses introduced by the RIL codons are probably overridden, increasing translation speed and consequently, protein aggregation (Rosano and Ceccarelli, 2009).

#### Limiting factors in batch cultivation

When the expression of the recombinant protein is low and cannot be increased by the proposed mechanisms, then the volumetric yield of desired protein can be augmented by growing the culture to higher densities. This can be achieved by changing a few parameters, like medium composition and providing better aeration by vigorous shaking (McDaniel and Bailey, 1969; Cui et al., 2006; Blommel et al., 2007).

LB is the most commonly used medium for culturing *E. coli*. It is easy to make, it has rich nutrient contents and its osmolarity is optimal for growth at early log phase. All these features make it adequate for protein production and compensate for the fact that it is not the best option for achieving high cell density cultures. Despite being a rich broth, cell growth stops at a relatively low density. This happens because LB contains scarce amounts of carbohydrates (and other utilizable carbon sources) and divalent cations (Sezonov et al., 2007). Not surprisingly, increasing the amount of peptone or yeast extract leads to higher cell densities (Studier, 2005). Also, divalent cation supplementation (MgSO_4_ in the millimolar range) results in higher cell growth. Adding glucose is of limited help in this regard because acid generation by glucose metabolism overwhelms the limited buffer capacity of LB, at least in shake flasks where pH control can be laborious (Weuster-Botz et al., 2001; Scheidle et al., 2011). If culture acidification poses a problem, the media can be buffered with phosphate salts at 50 mM. 2xYT, TB (Terrific Broth) and SB (Super Broth) media recipes are available elsewhere and have been shown to be superior to LB for reaching higher cell densities (Madurawe et al., 2000; Atlas, 2004; Studier, 2005).

A major breakthrough in media composition came in 2005 by the extensive work of Studier. In that report, the concept of autoinduction was developed (Studier, 2005). In autoinduction media, a mixture of glucose, lactose, and glycerol is used in an optimized blend. Glucose is the preferred carbon source and is metabolized preferentially during growth, which prevents uptake of lactose until glucose is depleted, usually in mid to late log phase. Consumption of glycerol and lactose follows, the latter being also the inducer of *lac*-controlled protein expression. In this way, biomass monitoring for timely inducer addition is avoided, as well as culture manipulation (Studier, 2014).

As the number of cells per liter increases, oxygen availability becomes an important factor with profound influence on growth (O’Beirne and Hamer, 2000; Losen et al., 2004).Oxygen limitation triggers the expression of more than 200 genes in an attempt to adjust the metabolic capacities of the cell to the availability of oxygen, all of which hinder optimal growth over long culture periods (Unden et al., 1995). The easiest way to increase the amount of available oxygen in shake vessels is to increase shaking speed. For regular flasks, the optimal shaking speed range is 400–450 rpm. More agitation is generated in baffled flasks; under these conditions, 350–400 rpm are enough for good aeration. However, vigorous shaking can induce the formation of foam, which will lower oxygen transfer. For this reason, the addition of an antifoaming agent is recommended, although it was shown that antifoams can affect the growth rate of several microorganisms and the yield of recombinant protein (Routledge et al., 2011; Routledge, 2012). Also, proper aeration depends on the ratio of culture volume to vessel capacity. As a rule of thumb, the culture volume should be less or equal to 10% of the shaking flask capacity, although in our hands, protein production with culture volumes occupying 20% of the flask capacity was possible (Rosano et al., 2011). A strategy that can produce significant increases in cell density is fed-batch fermentation. This approach has a wide availability of tools and methods, but it is beyond the scope of this paper and is addressed elsewhere (Yamanè, 1984; Yee and Blanch, 1992; Moulton, 2013).

Two rarely discussed parameters in the process of recombinant protein production are the preparation of the starting culture and the time of induction. Most protocols call for diluting a saturated overnight preculture (dilution factor 1/100) into the larger culture (Sivashanmugam et al., 2009). However, leaky expression of the chosen system can lead to plasmid instability, which may result in a poor yield of target protein. Also, in the starter culture, cells can be in dissimilar metabolic states. Upon dilution into fresh media, cells will grow at different rates leading to irreproducible induction points (Huber et al., 2009). A proper preculture (cells in an active equalized growing phase) can be prepared by growing the overnight starter culture at 20–25°C or by using a slow-release system for glucose, among other methods (Busso et al., 2008; Huber et al., 2009; Sivashanmugam et al., 2009). After inoculation and further growth, the inducer is often added in mid-log phase because the culture is growing fast and protein translation is maximal. However, induction at early stationary phase is also possible (Ou et al., 2004). In fact, in some cases the target protein was more soluble when inducer was added at this stage (Galloway et al., 2003). Presumably, the reduced rate of protein synthesis may result in less aggregation in IBs, as we describe below.

### INCLUSION BODIES FORMATION

When a foreign gene is introduced in *E. coli*, spatio-temporal control of its expression is lost. The newly synthesized recombinant polypeptide is expressed in the microenvironment of *E. coli*, which may differ from that of the original source in terms of pH, osmolarity, redox potential, cofactors, and folding mechanisms. Also, in high level expression, hydrophobic stretches in the polypeptide are present at high concentrations and available for interaction with similar regions. All of these factors lead to protein instability and aggregation (Hartley and Kane, 1988; Carrio and Villaverde, 2002). These buildups of protein aggregates are known as IBs. IB formation results from an unbalanced equilibrium between protein aggregation and solubilization. So, it is possible to obtain a soluble recombinant protein by strategies that ameliorate the factors leading to IB formation (Carrio and Villaverde, 2001, 2002). One is to fuse the desired protein to a fusion partner that acts as a solubility enhancer. Some examples were already described in Section “Affinity Tags.” In some cases the generation of IB can be an advantage, especially if the protein can be refolded easily *in vitro*. If that is the case, conditions can be adjusted to favor the formation IB, providing a simple method for achieving a significant one-step purification of the expressed protein (Burgess, 2009; Basu et al., 2011).

#### Disulfide bond formation

For many recombinant proteins, the formation of correct disulfide bonds is vital for attaining their biologically active three-dimensional conformation. The formation of erroneous disulfide bonds can lead to protein misfolding and aggregation into IB. In *E. coli*, cysteine oxidation takes places in the periplasm, where disulfide bonds are formed in disulfide exchange reactions catalyzed by a myriad of enzymes, mainly from the Dsb family (Messens and Collet, 2006). By contrast, disulfide bond formation in the cytoplasm is rare, maybe because cysteine residues are part of catalytic sites in many enzymes. Disulfide bond formation at these sites may lead to protein inactivation, misfolding, and aggregation (Derman et al., 1993). The cytoplasm has a more negative redox potential and is maintained as a reducing environment by the thioredoxin–thioredoxin reductase (trxB) system and the glutaredoxin–glutaredoxin reductase (gor) system (Stewart et al., 1998). This situation has a huge impact in the production of recombinant proteins with disulfide bonds. One option would be to direct the protein to the periplasm, as we have discussed in Section “Protein Toxicity.”

Nevertheless, expression in the cytoplasm is still possible thanks to engineered *E. coli* strains that possess an oxidative cytoplasmic environment that favors disulfide bond formation (Derman et al., 1993). Worthy of mention are the Origami (Novagen) and SHuffle (NEB) strains. We described earlier the Origami^TM^ strain, as having a *trx*B^-^ *gor*^-^ genotype in the K-12 background (as this double mutant is not viable, a suppressor mutation in the *ahp*C gene is necessary to maintain viability; Bessette et al., 1999). Origami^TM^ is also available in the BL21(DE3) *lacY* (Tuner^TM^, Novagen) background. Addition of the pRARE plasmid for the extra advantage of correcting codon bias resulted in the construction of the Rosetta-gami^TM^ B strain (Novagen). The SHuffle^®^ T7 Express strain [BL21(DE3) background, NEB] goes a little bit further. Besides the *trx*B^-^ and *gor*^-^ mutations, it constitutively expresses a chromosomal copy of the disulfide bond isomerase DsbC (Lobstein et al., 2012). DsbC promotes the correction of mis-oxidized proteins into their correct form and is also a chaperone that can assist in the folding of proteins that do not require disulfide bonds. Due to the action of DsbC, less target protein aggregates into IB.

#### Chaperone co-expression/chemical chaperones and cofactor supplementation

Molecular chaperones lie at the heart of protein quality control, aiding nascent polypeptides to reach their final structure (Hartl and Hayer-Hartl, 2002). Other specialized types of chaperones, like ClpB, can disassemble unfolded polypeptides present in IB. The high level expression of recombinant proteins results in the molecular crowding of the cytosol and quality control mechanisms may be saturated in this situation (Carrio and Villaverde, 2002). One strategy for solving this problem is to stop protein expression by inducer removal after a centrifugation step and addition of fresh media supplemented with chloramphenicol, an inhibitor of protein synthesis. This allows recruitment of molecular chaperones to aid in the folding of newly synthesized recombinant polypeptides (Carrio and Villaverde, 2001; de Marco and De Marco, 2004).

Given their function, it is not surprising that efforts to inhibit IB formation were directed to the co-expression of individual or sets of molecular chaperones (Caspers et al., 1994; Nishihara et al., 2000; de Marco et al., 2007). Commercially, one of the most used systems is the chaperone plasmid set from Takara (Nishihara et al., 1998, 2000). This set consists of five plasmids (pACYC derivatives) which allow overexpression of different chaperones or combinations of them: (i) GroES-GroEL, (ii) DnaK/DnaJ/GrpE, (iii) (i) + (ii), (iv) trigger factor, (v) (i) + (iv). On the other hand, if such a system is not at hand, the natural network of chaperones can be induced by the addition of benzyl alcohol or heat shock, though the latter is not recommended (de Marco et al., 2005).

When proteins are purified from IB, urea-denatured and then refolded *in vitro*, addition of osmolytes (also called chemical chaperones) in the 0.1–1 M range of concentration increases the yield of soluble protein (Rudolph and Lilie, 1996; Clark, 1998; Tsumoto et al., 2003; Alibolandi and Mirzahoseini, 2011). This situation can be mimicked *in vivo* by supplementing the culture media with osmolytes such as proline, glycine-betaine, and trehalose (de Marco et al., 2005). Also, the folding pathways that lead to the correct final conformation and stabilization of the proper folded protein may require specific cofactors in the growth media, for example, metal ions (such as iron-sulfur and magnesium) and polypeptide cofactors. Addition of these compounds to the batch culture considerably increases the yield as well as the folding rate of soluble proteins (Sorensen and Mortensen, 2005).

#### Slowing down production rate

Slower rates of protein production give newly transcribed recombinant proteins time to fold properly. This was previously addressed when we discussed the role of translational pauses at rare codons and their impact in the production of recombinant proteins. Moreover, the reduction of cellular protein concentration favors proper folding. By far, the most commonly used way to lower protein synthesis is reducing incubation temperature (Schein and Noteborn, 1988; Vasina and Baneyx, 1997; Vera et al., 2007). Low temperatures decrease aggregation, which is favored at higher temperatures due to the temperature dependence of hydrophobic interactions (Baldwin, 1986; Makhatadze and Privalov, 1995; Schellman, 1997).

When IB formation is a problem, recombinant protein synthesis should be carried out in the range 15–25°C, though one report described successful expression at 4°C for 72 h (San-Miguel et al., 2013). However, when working at the lower end of the temperature range, slower growth and reduced synthesis rates can result in lower protein yields. Also, protein folding may be affected as the chaperone network may not be as efficient (McCarty and Walker, 1991; Mendoza et al., 2000; Strocchi et al., 2006). The ArticExpress^TM^ (Stratagene) strain (B line) possesses the cold-adapted chaperonin Cpn60 and co-chaperonin Cpn10 from the psychrophilic bacterium *Oleispira antarctica* (Ferrer et al., 2004). The chaperonins display high refolding activities at temperatures of 4–12°C and confer an enhanced ability for *E. coli* to grow at lower temperatures (Ferrer et al., 2003).

### PROTEIN INACTIVITY

Obtaining a nice amount of soluble protein is not the end of the road. The protein may still be of bad quality; i.e., it does not have the activity it should. Incomplete folding could be the culprit in this scenario (Gonzalez-Montalban et al., 2007; Martinez-Alonso et al., 2008). In this case, the protein adopts a stable soluble conformation but the exact architecture of the active site is still unsuitable for activity. Some options already addressed can be helpful in these cases. Some proteins require small molecules or prosthetic groups to acquire their final folded conformation. Adding these compounds to the culture media can increase the yield and the quality of the expressed protein significantly (Weickert et al., 1999; Yang et al., 2003). Also, erroneous disulfide bond formation can lead to protein inactivity (Kurokawa et al., 2000). In addition, protein production at lower temperatures has a profound impact on protein quality. Work by the Villaverde lab has shown that conformational quality and functionality of highly soluble recombinant proteins increase when the temperature of the culture is reduced (Vera et al., 2007). This was also the case when the intracellular concentration of the chaperone DnaK was elevated (Martinez-Alonso et al., 2007). This phenomenon calls into question the use of solubility as an indicator of quality. Based on this fact, then it may be wise to express all recombinant proteins at low temperatures or at least, to compare the specific activity of a recombinant protein obtained at different temperatures.

If the activity of the heterologous protein is toxic to the cell, genetic reorganization of the expression vector leading to loss of activity may occur, allowing the host to survive and eventually take over the culture (Corchero and Villaverde, 1998). This structural instability of the plasmid can be detected by DNA sequencing after purification of the plasmid at the end of process. Any point mutation, deletion, insertion, or rearrangement may explain the low activity of a purified recombinant protein (Palomares et al., 2004).

## CONCLUDING REMARKS

In terms of recombinant expression, *E. coli* has always been the preferred microbial cell factory. *E. coli* is a suitable host for expressing stably folded, globular proteins from prokaryotes and eukaryotes. Even though membrane proteins and proteins with molecular weights above 60 kDa are difficult to express, several reports have had success in this regard (our laboratory has produced proteins from plants in the 90–95 kDa range; Rosano et al., 2011). Large-scale protein expression trials have shown that <50% of bacterial proteins and <15% of non-bacterial proteins can be expressed in *E. coli* in a soluble form, which demonstrates the versatility of the system (Braun and LaBaer, 2003). However, when coming across a difficult-to-express protein, things can get complicated. We hope to have given a thorough list of possible solutions when facing the challenge of expressing a new protein in *E. coli*. Nevertheless, a word of caution is needed. Many of the approaches described in this review will fail miserably in a lot of cases. This can be explained by the fact that strategies aiming at troubleshooting recombinant protein expression are sometimes protein specific and suffer from positive bias; i.e., things that work get published, all the others, do not. That being said, thanks to the efforts of the scientific community, the general methods available in the literature are no longer anecdotal and can be used systematically. Moreover, the field is always expanding and even after almost 40 years from the first human protein obtained in *E. coli* (Itakura et al., 1977), there is still much room for improvement.

## AUTHOR CONTRIBUTIONS

Germán L. Rosano and Eduardo A. Ceccarelli wrote the manuscript and approved its final version.

## Conflict of Interest Statement

The authors declare that the research was conducted in the absence of any commercial or financial relationships that could be construed as a potential conflict of interest.
